# Supplementary material for: Impact of a Teacher Intervention to Encourage Students to Eat School Lunch
Source: Int J Environ Res Public Health. 2022 Sep 14;19(18):11553. doi: 10.3390/ijerph191811553 (PMC9517446; doi:10.3390/ijerph191811553)
Supplement: Supplementary file 1 [file ijerph-19-11553-s001.zip › ijerph-1887379-supplementary.pdf]

SFUSD School Nutrition

School Staff and Teacher Questionnaire

Dear Teachers,

SFUSD has been working to change the student dining experience. Researchers at the University of California are studying the impact of these changes on students and staff. We would like your input about these changes and hope you will complete this questionnaire. It should take about 5 minutes of your time.

- Your answers to this questionnaire are anonymous and your participation is voluntary.
- The findings about your school will be kept confidential. Only aggregate (all schools) results will be shared and your school's name will not be identified if individual school findings are presented.
- Please submit the completed survey to the front office along with the student surveys completed in your classroom.
- If you choose to submit your contact information separate from the survey you will be entered into a raffle for $100 gift card to Amazon. Please see instructions on the attached sheet if you wish to be entered into the raffle.

Your input is extremely valuable. We appreciate your time!!

Sincerely,
Student Nutrition Services

Instructions: Please fill in a circle for each question.

School food

1. <u>This school year</u>, how often did you:

	Never	≤ 1 time per month	2-3 times per month	1-3 times per week	≥ 4 times per week
a. Eat the school lunch?	❍	❍	❍	❍	❍
b. Purchase **<u>food items</u>** from student vending machines at your school?	❍	❍	❍	❍	❍
c. Purchase **<u>food items</u>** from faculty/staff vending machines at your school?	❍	❍	❍	❍	❍
d. Purchase **<u>beverages</u>** from student vending machines at your school?	❍	❍	❍	❍	❍
e. Purchase **<u>beverages</u>** from faculty/staff vending machines at your school?	❍	❍	❍	❍	❍
f. Purchase **<u>food items</u>** from school dining?	❍	❍	❍	❍	❍
g. Purchase **<u>beverages</u>** from school dining?	❍	❍	❍	❍	❍

2. How strongly do you agree with the following statements:

	Strongly Disagree	Disagree	Agree	Strongly Agree	N/A
a. School meals are healthy.	❍	❍	❍	❍	❍
b. School meals taste good.	❍	❍	❍	❍	❍
c. Students think the school meals taste good.	❍	❍	❍	❍	❍

About you

3. How strongly do you agree with the following statements:

	Strongly Disagree	Disagree	Unsure	Agree	Strongly Agree
a. I consider myself in good to excellent health.	❍	❍	❍	❍	❍
b. I am satisfied with my own eating habits.	❍	❍	❍	❍	❍

About your students

4. <u>This school year</u>, how often did you:

	Never	≤ 1 time per month	2-3 times per month	1-3 times per week	≥ 4 times per week
a. Discuss healthy eating with your students?	❍	❍	❍	❍	❍
b. Discuss the importance of exercise with your students?	❍	❍	❍	❍	❍
c. Encourage your students to make healthy **food** choices?	❍	❍	❍	❍	❍
d. Encourage your students to make healthy **beverage** choices?	❍	❍	❍	❍	❍
e. Encourage your students to eat the school lunch?	❍	❍	❍	❍	❍
f. Eat lunch with your students in the cafeteria?	❍	❍	❍	❍	❍
g. Eat lunch in the classroom with your students?	❍	❍	❍	❍	❍
h. Eat fruits or vegetables in front of students?	❍	❍	❍	❍	❍
i. Drink water in front of students?	❍	❍	❍	❍	❍
j. Drink soda or other sugar-sweetened beverages in front of students?	❍	❍	❍	❍	❍

Thank you for your input!
